# Effective Visualization and Easy Tracking of Extracellular Vesicles in Glioma Cells

**DOI:** 10.1186/s12575-019-0092-2

**Published:** 2019-03-15

**Authors:** Abir Mondal, K. A. Ashiq, Prashant Phulpagar, Divya Kumari Singh, Anjali Shiras

**Affiliations:** National Centre for Cell Science (NCCS), Savitribai Phule Pune University Campus, Pune, India

**Keywords:** Extracellular vesicles, Immunolabeling, Cellular uptake, Visualization, Tracking, Angiogenesis

## Abstract

Extracellular vesicles (EVs) are nano-sized, membrane-bound structures secreted by cells and play critical roles in mediating intercellular signaling. EVs based on their size as well as mechanisms of biosynthesis are categorized as either microvesicles (200–1000 nm) or exosomes (30–200 nm). The EVs carry several biomolecules like proteins, DNAs, RNAs, and lipids into other cells and modulate several cellular functions. Being of very small sizes, it is very challenging to analyze them using conventional microscopes. Here, we report a new method developed by us for visualizing EVs using simple immune-fluorescence based technique, wherein the isolated EVs can be stained with fluorescently tagged antibodies to proteins present in EVs. The stained EVs can then be analyzed by using either confocal or super-resolution microscopes. Our method detailed here is equally effective in staining proteins that are present inside the EVs as well as those localized to the membranes of vesicles. By employing unique staining strategies, we have minimized the background noise and thereby improved the signal strength in confocal microscope. Using electron microscopy, we have ascertained that the structural integrity of the labeled EVs is intact. More importantly, the labeling of EVs does not affect their functionality and their localization can be tracked after its uptake by recipient cells without resorting to any conventional reporter-based strategies or lipophilic dyes. In conclusion, the method described here is a simple, sensitive and efficient immune-fluorescence based method for visualization of molecules within the EVs.

## Introduction

Cells of multicellular organisms communicate with each other through varied mechanisms. A prominent way is through exchange of information through biomolecules secreted by cells followed by their uptake by neighboring recipient cells. For their effective uptake, the secreted molecules are packaged into small membrane bound vesicles known as extracellular vesicles (EVs). These EVs have an average diameter of around 30–1000 nm [1] and are further classified based on their size and biosynthetic pathway as exosomes (30–200 nm) or microvesicles (200–1000 nm) [2–4]. The microvesicles are formed by outward budding and fission of the cell membrane, whereas exosomes are released from the cells by invagination of the cell membrane followed by their exocytosis [2]. The EVs express molecules like CD63, CD81, CD9, HSP70 etc. [3]. Almost all cells secrete EVs and these EVs have the potential to modulate various cellular functions under both physiological and pathophysiological conditions [5]. The EVs have been associated with diverse functions that include cell growth, proliferation, angiogenesis, metastasis and therapy resistance [6–8]. Whilst, their participation in each of these complex processes is being analyzed, their specific role in each of these pathways in relation to their functional involvement in various signaling pathways requires detailed elucidation. The EVs due to their complex nature and very small size are difficult to characterize and furthermore tough to view. Use of advanced methodologies that include electron microscopy and atomic force microscopy are useful in their characterization. However, the complex nature of these methodologies precludes their use and necessitates development of simple protocols for their visualization.

Till date, there are very few methods available for fluorescent labeling of EVs isolated from cell cultures and their direct visualization using immune-fluorescence microscopy. The previously described methods are based on isolation of EVs from cells and their labeling using dyes like - PKH, Did, CFSE for their visualization and in vitro tracking in recipient cells [9–11]. PKH dyes are one of the most widely used lipophilic dyes for EV labeling [12, 13]. However, most of these lipophilic dyes often label EVs non-specifically as other cellular components also get labeled and thereby generate false positive signals [14, 15]. Since, PKH dyes are very stable, the unbound dyes get retained in the cells, thereby producing background signals in cellular uptake assays by staining of recipient cells. In summary, lipophilic dyes are not reliable EV labeling agents unless one has an entirely pure population of EVs that is completely devoid of cellular components [16].

Another way to label EVs, is by cloning the EV specific markers (like CD63, CD9, CD81) in reporter vectors that are tagged with GFP/RFP followed by their transfection into cells of interest [17–20]. However, there are limitations in using fluorescent protein conjugated EV labeling methods. Specifically since, EV signatures are not similarly expressed in EVs derived from all cell-types and they very often show heterogeneity in similar or different cell-types [21]. Thus, the use of reporters conjugated to proteins enriched in EVs, is only restricted to subpopulations of EVs. This limits their wider use in observing multiple EV types without the use of single EV analysis (SEA) technology [21]. Unfortunately, genetic labeling cannot be performed with plasma EVs such as isolates from human blood.

There are very few methods available for staining of specific biomolecules present on EVs and most are based on tetraspanin proteins such as CD63, CD9, CD81 etc. This restricts the detection of only surface markers of EVs. Additionally, these methods require immobilization of EVs on a slide coated with antibody or avidin-biotin based microfluidic device for staining and visualization via confocal and stimulated emission depletion (STED) microscopy [21, 22].

Here, we report development of a novel, elegant, simple and quick method for labeling of EVs mainly exosomes and micro-vesicles in a cell-free system using immune-fluorescence strategy followed by their visualization using confocal microscopy. In this procedure, we isolate EVs from the cells and stain them with antibodies to intra-vesicular proteins such as TSG101 and HSP70 which are enriched in these organelles and examine them using immune-fluorescence methods. Importantly, this procedure is helpful for study of any protein, that one needs to analyze from the EVs and is as simple as detecting any cellular protein by immune-fluorescence (IF) labeling procedure. As a proof of principle, we have used surface markers like CD63 as well as inter-vesicular markers like TSG101 and HSP70 to label the EVs in a microfuge tube. Interestingly, the labeled EVs are efficiently internalized by the recipient cells and these can be easily tracked upon their internalization. We here demonstrate this labeling procedure with a patient derived glioblastoma (GB) cell-line KW10, but our method is effective for any kind of tumor derived cell-lines from any tumor type.

## Methods

### Cell Culture

The KW10 cell-line used in this study was developed by us and we have extensively characterized it as a patient-derived glioblastoma cell-line [23]. As the fetal bovine serum (FBS) used for culturing most of the cell-lines is often contaminated with bovine EVs, we have performed ultra-centrifugation of the medium at 100,000 g for 18 h to get rid of them and thereby ensured that the medium was free of extraneous EVs. We cultured the GB cell-line KW10 in Dulbecco’s Modified Eagle Medium/Nutrient Mixture F-12 (DMEM/F12) with 10% FBS (EV depleted) and collected the Conditioned Medium (CM) after growth of cells in this medium for 48–72 h.

We have also cultured human umbilical vein endothelial cells (HUVECs) on fibronectin (Invitrogen # 33016015) coated plates with endothelial cell media (Sigma # 211–500) for their use in uptake and tube formation assays.

### Isolation and Characterization of EVs

The detailed steps are as follows:

A. **Conditioned media processing**
  The collected conditioned medium was centrifuged at 4 °C for 10 mins at 800 x g in a swinging bucket rotor (Eppendorf 5804R; rotor ID A-4-44) to remove cellular debris. Later, the conditioned media was filtered through a 0.45 μm syringe filter with supor membrane (Pall # 4654) and stored at − 80 °C for later use (maximum 1–2 months) or at 4 °C for immediate use.
B. **EV isolation**

- 20% PEG10000 (Sigma cat. - 92,897) solution was prepared with deionized water and passed through a 0.22 μ filter (Millipore # SLGP033RS) [24].
- 5 ml conditioned medium was taken in a 15 ml centrifuge tube and another 5 ml of 20% PEG10000 solution was added to it.
- The above solution was mixed by inverting the tube 10 times and incubated on ice for 1 h.
- The mixture was centrifuged at 3000 x g for 30 min at 4 °C in a swinging bucket rotor (Eppendorf 5804R; rotor ID A-4-44). An off-white pellet was visible. The supernatant was removed carefully by decanting without disturbing the EV pellet. Another round of centrifugation was performed at 3000 x g for 5 mins to remove the residual liquid in the tube by pipetting.
- Collectively, the entire pellet was dissolved in 200 μl of PBS. The dissolved pellet was collected in a fresh 1.5 ml micro-centrifuge tube.
  [Note: 20 ml of conditioned media was used for protein labeling. Avoid keeping CM at − 20 °C because it may result in a meager yield during EV isolation. The use of freshly prepared conditioned medium is recommended to get higher yield during EV isolation. If the pellet is not visible, the volume of the conditioned media can be increased to obtain a larger pellet.]

C. **EV characterization by Western Blotting**
  EVs and cells were lysed using 1X RIPA buffer and 20 μg of protein was loaded into each well for western blotting. Next, we characterized the EVs by western blotting using antibodies to several EV specific markers like TSG101 (1:3000 dilution; Pierce - MA123296), CD63 (1:1000 dilution; Abcam - ab59479) and HSP70 (1:3000 dilution Cloud clone- MAA873Hu21). Cellular markers like - calnexin (1:2000 dilution; CST - 2679S) which are not expressed by EVs served as negative control. The secondary antibodies used were IRDye 680RD Goat anti-Mouse IgG (1:8000 Dilution; Licor 926–68,070), IRDye 800CW Goat anti-Rabbit IgG (1:8000 dilution; Licor 926–32,211) and Donkey anti-Goat IgG HRP (1:7000 dilution; Invitrogen - A15999) (Fig. 2a).

### Protocol for EV Immune-Labeling

**Fig. 1:**
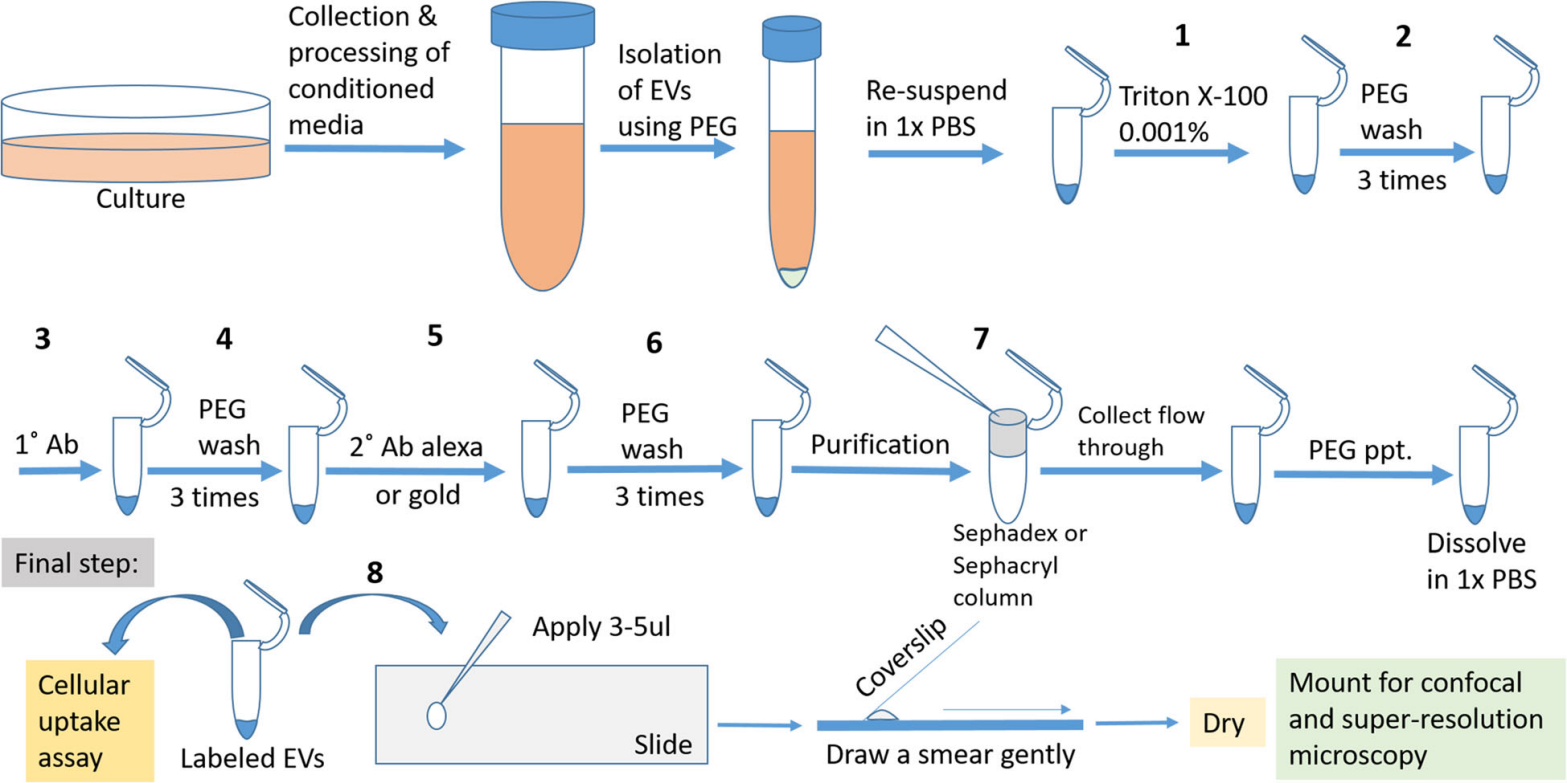
Strategy for labeling of Extracellular Vesicles (EV) derived from glioma cells. Flow chart detailing the steps involved in labeling of EVs in cell-free system

The flow-chart for EV immune-labeling is shown in Fig. 1. All labeling experiments were performed at least 7 times by various people in the lab and we achieved similar results. The steps followed for EV immune-labeling procedure are as follows.

1. The Isolated EVs Were Permeabilized Using 0.001% (Final Concentration) Triton X-100 for 5 Min.
  [Note: We tried different concentrations like- 0.01, 0.05 and 0.001% of Triton X-100; amongst them, we found that only 0.001% concentration of Triton X-100 was optimum for maintaining the integrity of EVs.]
2. PEG10000 was added to a final concentration of 10% as mentioned above (no incubation required) and the suspension was spun at 3000 x g at room temperature (RT) for 5 mins. The supernatant was carefully removed and the pellet was dissolved in 100 μl of PBS.
3. Primary antibodies to TSG101, CD63 and calnexin (final concentration of 0.01 μg/μl) were individually added to the dissolved EV suspension of KW10 cells. Likewise, 1 μg of each of these antibodies were individually added to 100 μl of EV preparation in a microfuge tube. The whole mixture was incubated for 90 mins with gentle shaking at RT.
4. Next, 100 μl of 20% PEG10000 solution was added to the EV suspension in step 3, and the whole mixture was centrifuged at 3000 x g for 5 mins. The obtained pellet was re-suspended in 100 μl of 1x PBS. The step of PEG precipitation of EVs was repeated twice to remove excess antibodies. The EV pellet was suspended in 100 μl of PBS.
5. Alexa Fluor labeled species specific secondary antibodies (Invitrogen Catalog no. A11005 and A11012) diluted 1:100 were added to the EV suspension and the complex was incubated for 1 h with gentle shaking in the dark.
  [Note: Primary and secondary antibody concentration may vary depending on the size of the pellet observed during EV isolation.]
6. The unbound secondary antibodies were washed of using PEG10000 at speed of 3000 x g thrice in a centrifuge as mentioned in step-4. The pellet was dissolved in 100 μl of PBS.
7. PEG can precipitate macromolecules. Hence, Sephadex G-25 column (G2580 Sigma) was used to remove the unbound antibodies and primary-secondary antibody conjugates which may give false positive signal. The solution was passed through the column to remove background noise. Then PEG solution was added to flow through for further precipitation of EVs. The mixture was centrifuged at 3000 x g for 5 mins and the supernatant was removed with a pipette. The pellet was dissolved in 20 μl of PBS.
8. 3–4 μl of dissolved pellet of each labeled EV-antibody complex was placed on pre-cleaned microscope slides. A thin smear was drawn using coverslip and kept for drying in dark for 5–10 min. Further the slides were analyzed using confocal and STED microscopy (Leica TCS SP8 STED 3X). We also performed dSTORM  imaging on Nanoimager S (ONI Oxford). [Note: We used anti-mouse secondary antibodies Alexa 488 (Invitrogen A11001) and Alexa 555 (Invitrogen A21422) for dSTORM imaging.]

[Note: We tried different columns to remove background noise. Sephadex G-25 showed 80–90% efficiency in eliminating background noise. Sephacryl (range 20–8000 KDa) worked best for removing unbound primary-secondary antibody conjugates but gave a very low yield. Data provided here was generated using Sephadex G-25 column. Column packing must be done carefully and air bubbles must be strictly avoided inside the column].

To prove that the method of detecting EVs by immunofluorescence is as sensitive to the methods that are based on detection of EVs by PKH67 labeling, we performed a dual labeling experiment, wherein upon antibody labeling of EVs, we also labeled the same EVs using PKH67 dyes as well.

### Dual Labeling with PKH67 Dye

- After completing the secondary antibody staining at step-5 (in above), unbound secondary antibody from each reaction was washed using PEG10000 as mentioned above. After the 3rd wash, the pellet was dissolved in 50 μl of PKH diluent and 0.2 μl of PKH67 (PKH67GL Sigma) was added to it.

- The mixture was incubated at RT for 30 mins with gentle shaking.

- 50 μl of 20% PEG10000 solution was added to the mixture for the EV precipitation. Then the mixture was centrifuged at 3000 x g for 5 mins. The supernatant was removed very carefully and the EVs were suspended in 50 μl of PBS.
  [Note: You may not get a visible pellet here if you begin with less than 20 ml of CM].

- The mixture was passed through the column (Sephadex G25). Flow through was collected and 100 μl of 20% PEG10000 was added to it. The mixture was centrifuged at 3000 x g for 10 mins (visible pellet may not be seen). The supernatant was removed very carefully and 10 μl of PBS was added to it.
  [Note: Sephadex G25 column is not efficient to remove unbound PKH dye].

- Finally, 3–4 μl of dissolved pellet was placed on pre-cleaned slide. A thin smear was drawn as mentioned above (step 8) and mounted for visualization by confocal microscopy (Leica TCS SP5 II).

Next, we checked the integrity of the EVs by transmission electron microscopy (TEM) using gold labeled secondary antibodies.

### Sample Preparation for TEM

To perform gold labeling of EVs, we replaced Alexa Fluor labeled secondary antibody with gold nanoparticle tagged secondary antibodies (anti-mouse gold IgG, sigma G7527 and anti-rabbit gold IgG, Sigma G7402) and followed a similar immune-staining protocol as described above. To observe the immuno-gold labeled EVs under TEM, we processed our samples using the uranyl acetate staining protocol as described below.

#### Reagents Required

2% paraformaldehyde in PBS, 4% uranyl acetate (pH 4) and 2% methylcellulose in filtered and deionized water.

#### Working Solution

1 volume of 4% uranyl acetate was mixed with 9 volumes of 2% methylcellulose and the solution was kept in the dark.

- 3 to 5 μl of labeled and unlabeled EVs were applied on copper grids (Ted Pella Prod# 01810) respectively and incubated for 30 mins to settle at RT.
- 2% paraformaldehyde (PFA) solution was applied on parafilm. The grids were placed inversely on the drop of 2% PFA solution for fixation of the sample and incubated further for 20 mins in RT.
- Similarly, few drops of deionized water was applied on parafilm. Then, grids were placed on water drop to remove extra paraformaldehyde without any incubation (Note: PFA solution was prepared in PBS. PBS can precipitate uranyl acetate, hence washing step is necessary to obtain proper resolution of the samples).
- A 90 mm petri-dish was taken and parafilm was kept inside the petri-dish. Later, few drops of freshly prepared working solution was added on the top of parafilm. The whole preparation was kept on ice (in dark). Finally, grids were placed on the drop of working solution and incubated for 10 mins (on ice and in dark condition).

- Grids were removed by using clean fine forceps and extra solution was soaked using blotting paper.
- Finally, grids were kept in special grid holder. This was followed by imaging using TEM (FEI Tecnai T20). Images were taken at 100 kV.

### Tracking of EVs in Recipient Cells

- At a final step of EV labeling, the labeled EV pellet was dissolved either in 20 μl of endothelial medium or in sterile PBS.

- The EVs were incubated for 6 h on HUVECs (Invitrogen cat no. C0035C) which were pre-seeded on the coverslips.

- The coverslips were washed with PBS thrice and fixed with 4% PFA solution for 5 mins.

- Further, the coverslips were washed 3 times using PBS and incubated with 1 μg/ml DAPI (Invitrogen #D1306) for 10 mins in dark.

- Next, the coverslips were washed 3 times using PBS and finally mounted on slides for confocal imaging (Leica TCS SP5 II). Images were acquired using 63X oil immersion lens.

### Angiogenesis Assay for Checking Functionality of EVs

Angiogenesis assay was performed according to the method of Guo et al. [25]. 1 × 10^4^ number of HUVECs were plated on the growth factor reduced Matrigel (Corning product # 354230) in μ-slide (from ibidi cat no. 81506) in three different conditions that included – negative control, unlabeled EVs and labeled EVs. Then the slides were incubated at 37 °C in CO_2_ incubator for 6 h. Later, the cells were analyzed for their tube forming potential using AngioTool software of National Institute of Health (NIH) [26]. We have performed analysis of every image under similar configuration of AngioTool64 software version 0.6a (02.18.14).

### Statistical Analysis

Statistical analysis was conducted using GraphPad Prism 5 software. Two-tailed t-test (Mann-Whitney U) was performed for analyses of size distribution of EVs. Bars in all figures represent mean ± SEM.

## Results

### Labeling of EVs Using CD63, TSG101, HSP70 and Calnexin Antibodies

**Fig. 2:**
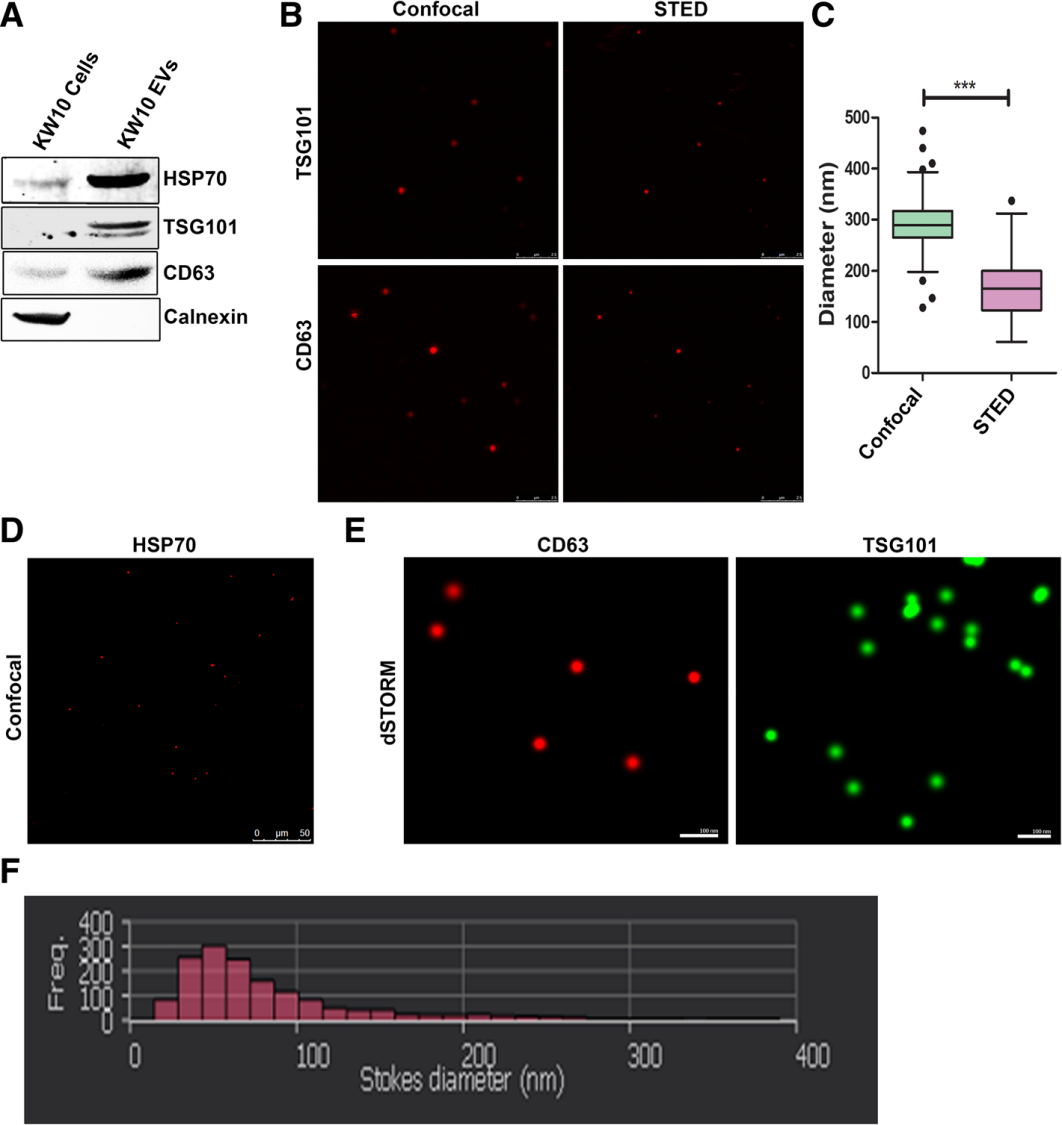
Characterization and visualization of EVs using confocal and STED microscope. Western blotting for different exosomal marker proteins like- TSG101, CD63, HSP70 and ER marker like- calnexin (**a**). Confocal and STED imaging of EVs labeled with TSG101 and CD63 antibodies and visualized using Alexa 594 species specific antibodies (**b**). Analyses and comparison of the size of EVs through confocal and STED microscopy (p*** < 0.0001) (**c**). Confocal imaging for HSP70 labeled EVs (**d**). dSTORM images of EVs stained with CD63 (red) and TSG101 (green) antibodies (**e**). Analyses of the size distribution of immune-labeled EVs by dSTORM Nanoimager (**f**)

We performed EV characterization by western blotting (Fig. 2a). The EVs derived from KW10 cells expressed all the EV specific markers like CD63, TSG101 and HSP 70 but distinctly lacked expression of endoplasmic reticulum (ER) marker calnexin confirming the specificity of the EV isolation procedure. Next, we used the EV immunostaining approach wherein EVs were analyzed for expression of specific surface marker proteins like CD63 and for presence of specific intra-vesicular proteins like TSG101 and HSP70. As shown in Fig. 2b, we obtained a very specific fluorescence signal for CD63 and TSG101 in the EVs by confocal staining and by STED (Fig. 2b). We used the Fiji-ImageJ software [27], to calculate the size of EVs and found that it was in the range of 60 to 350 nm (Fig. 2c). Our data indicated that STED yielded a better size distribution as compared to the confocal microscope (*p* < 0.0001) and this was in line with the existing reports on EV size [22]. Another intra-vesicular protein HSP70 was also specifically detected by confocal imaging (Fig. 2d). We also performed dSTORM imaging to analyze expression of CD63 and TSG101 (Fig. 2e) in the vesicles using Nanoimager S (ONI Oxford). With this imager we found that majority of labeled vesicles were distributed between 30 to 250 nm sizes (Fig. 2f). The calnexin staining on the Nanoimager S (dSTORM) gave a signal corresponding to a particle size of around 2 to 15 nm which did not correlate with the size of EVs. We made this signal as a background, and then analyzed fluorescent signals only from those vesicles which were labeled by TSG101 and CD63 respectively (on different slide). Thus, by following this signal elimination step we were able to successfully label both the surface and intra-vesicular proteins of EVs derived from glioma cells.

### Co-Localization of Labeled EVs with Conventional PKH67 Dye

**Fig. 3:**
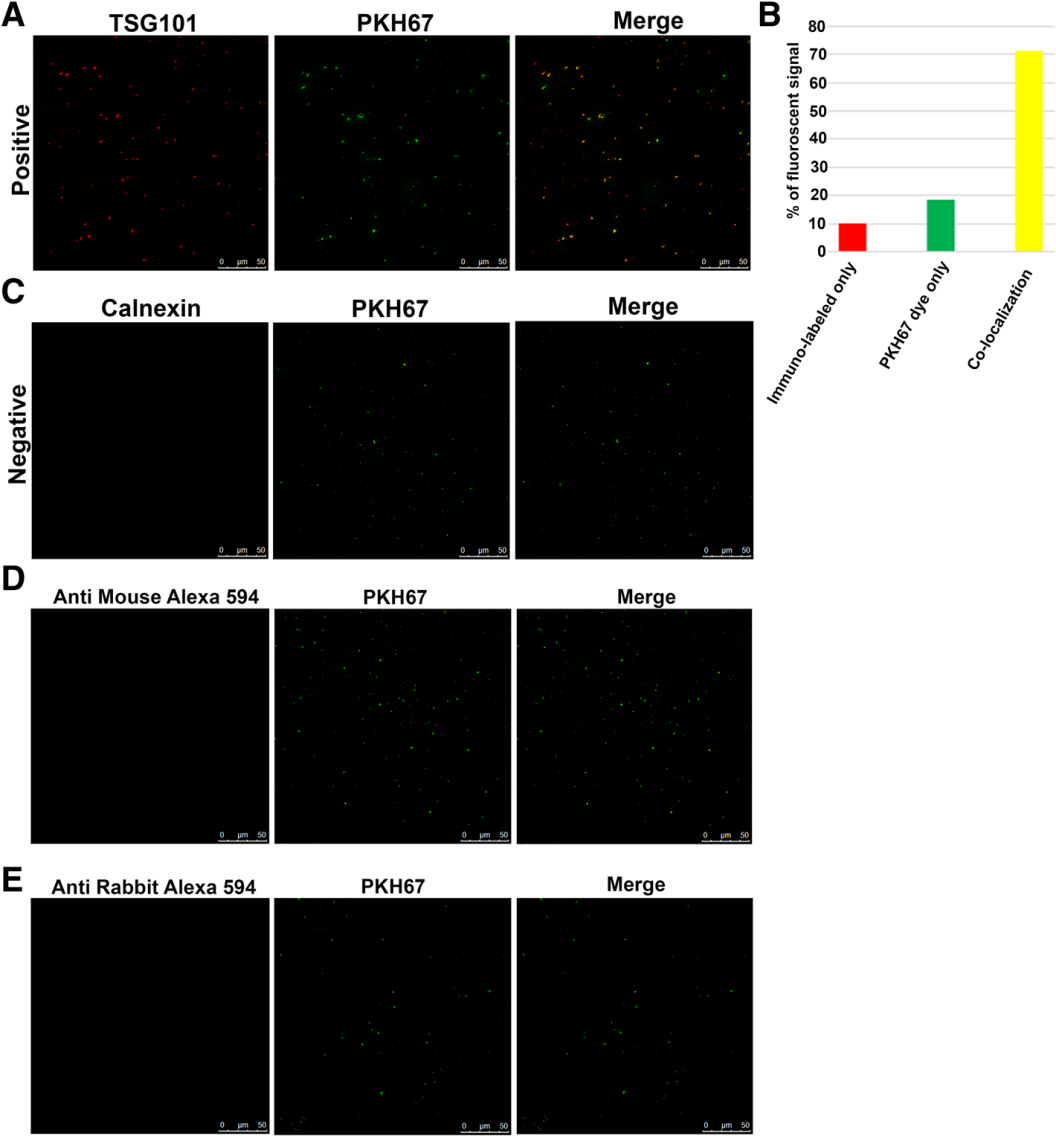
Co-localization of EVs stained with TSG101 and calnexin with PKH67. Confocal imaging of PKH67 (green) dye stained the EVs that were immune-labeled using TSG 101 antibody (red). The PKH67 dye (green) got co-localized with TSG101 and yielded yellow signal (merge) (**a**). Analyses of the percentage of fluorescent signal observed from red (immuno-labeled only), green (PKH67 dye only) and yellow (co-localization of both) channel (**b**). PKH67 dye retained fluorescent signal whereas calnexin staining was negative in case of dual labeled EVs (**c**). Confocal imaging of EVs treated with secondary antibodies and PKH67 dye (**d** and **e**)

Specificity of labeling is a major limitation for EV visualization and for performing functional assays with EVs. To verify the specificity of labeling, we performed dual labeling procedure in which the EVs were stained with both EV specific antibody and a conventional PKH67 dye. We found that PKH67 dye co-localized with the pre-labeled TSG101 molecules in EVs which further strengthened our developed staining method (Fig. 3a). Next, we calculated the percentage of fluorescent signal obtained by our method by manually counting number of red, green and yellow spots (Fig. 3b). Surprisingly, about 10% of immuno-labeled (red signal) EVs did not get co-stained with PKH67 dye. Instead, 18% of the green fluorescent signal was observed from PKH67 dye, which supported the existing data on non-specificity of lipophilic labeling [14, 15]. Interestingly, we found that more than 70% of antibody labeled EVs got co-localized with PKH67 dye. Additionally, we also used pre-calnexin stained EVs for PKH67 dye labeling. As expected, the EVs got labeled with PKH67 dye but there was no signal for calnexin by confocal microscopy (Fig. 3c). Similar results were observed in case of EVs labeled with secondary antibody and PKH67 dye (Fig. 3 d, e). Thus, our method yielded a very specific signal to the EVs without generating any background noise.

### Integrity of EVs Using TEM

**Fig. 4:**
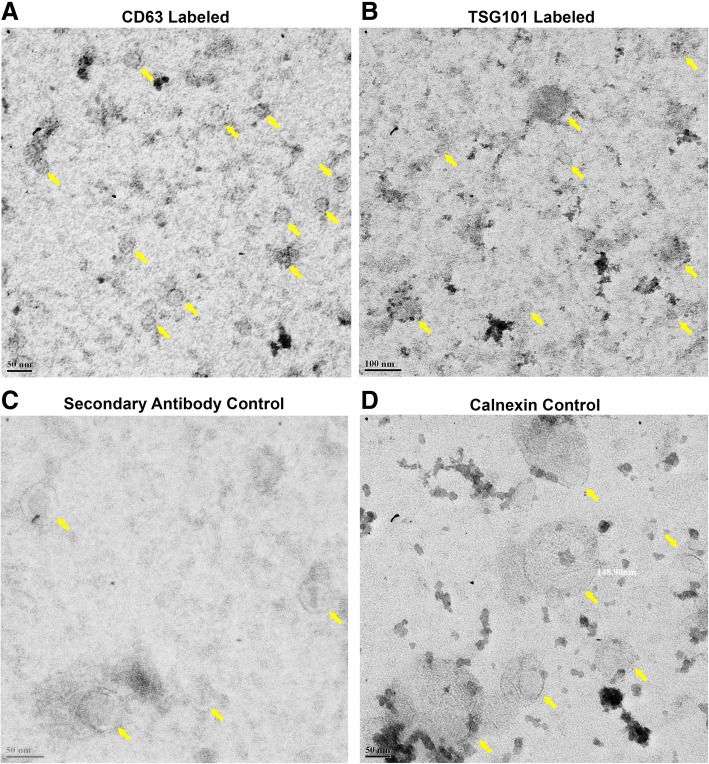
Investigating the structural integrity of EVs after labeling. Transmission electron micrographs of both labeled and unlabeled EVs. CD63-gold label EVs showed black dot on the surface (**a**). TSG101 labeled EVs showed whole EV staining on EM (**b**). Electron micrograph of EVs treated with secondary antibody (**c**). Calnexin control labeling indicated specificity of labeling procedure (**d**). EVs were indicated using yellow arrow

We used PEG to precipitate EVs at every step of our protocol. Additionally, we also used Triton X-100 for the permeabilization of EVs. As a result, there was a possibility that the structural integrity of EVs may get affected. Hence, we investigated whether use of PEG and Triton X-100 was interfering with the integrity of EVs. For this, we used gold labeled secondary antibody for staining with CD63 antibody and observed for presence of labeled EVs by TEM. We found that our protocol did not affect the structure of EVs. We obtained large number of EVs with a diameter of 27 to 230 nm. From our data, it is evident that the CD63 molecules (visible black dot) were localized to the surface of EVs (Fig. 4a). There were some vesicles which were not labeled with CD63 and this could be a result of heterogeneity found in EV signatures in tumor cells. We obtained similar data for EVs which were labeled with TSG101 (Fig. 4b). We did not observe any gold labeling in EVs which were only treated with secondary gold antibody as control confirming the specificity of our approach (Fig. 4c). Similarly, we did not observe non-specific staining in case of EVs labeled with calnexin (Fig. 4 d) further ensuring the specificity of our labeling method.

### Cellular Uptake Assay

**Fig. 5:**
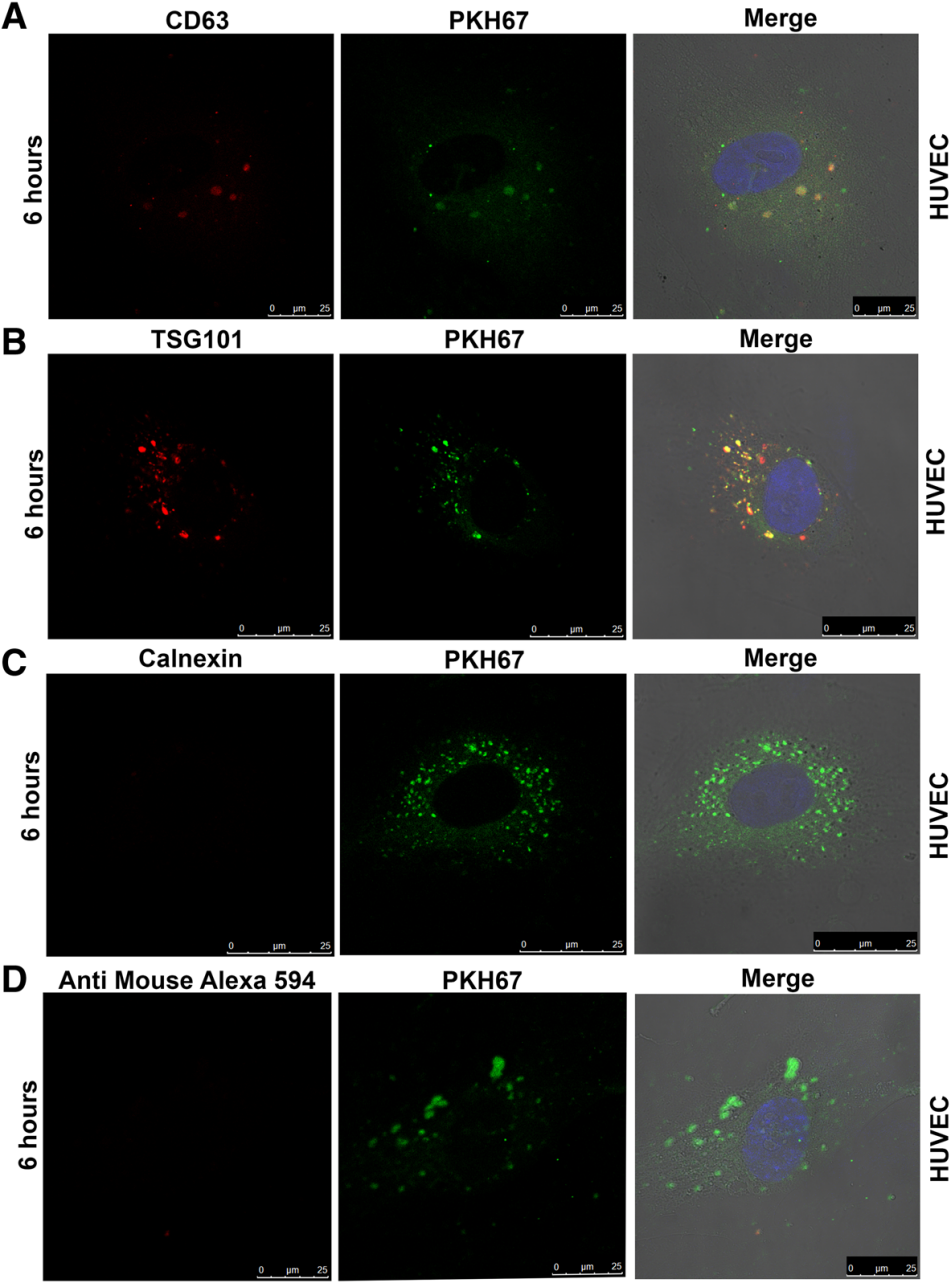
Cellular uptake assay in HUVECs. Confocal imaging of EVs labeled with antibodies to CD63, TSG101, calnexin along with PKH67 dye. The uptake of CD63 (upper panel) and TSG101 antibodies (lower panel) labeled EVs along with PKH67 dye molecules were analysed in HUVECs after 6 h of incubation with EVs (**a** and **b**). Calnexin and PKH67 dual staining served as a negative control to ensure specificity of signal (**c**). EVs labeled with only secondary antibody and PKH67 dye served as negative control (**d**)

Cellular uptake of EVs is a widely used assay to study the specificity of EVs to recipient cell types and for determining their function upon uptake. Tumor cell derived EVs majorly contribute to the formation of tumor vasculature. Considering the existing literature about the interaction of endothelial cells with tumor cells via EVs, we used endothelial cell-line (HUVEC) for the uptake assay [7, 28, 29]. After 6 h of incubation of dual labeled EVs with HUVECs, an effective uptake of EVs was detected in these cells (Fig. 5a and b). EVs labeled with either TSG101 and PKH67 or CD63 and PKH67 were effectively taken up by the HUVECs (Fig. 5a and b). Instead, the EVs which were stained with calnexin and PKH67 or secondary antibody and PKH67 showed fluorescent signal only to the PKH67 dye which further indicated that our protocol was very effective for studying cellular uptake of EVs (Fig. 5c and d).

### Labeled EVs with Potential Angiogenic Properties

**Fig. 6:**
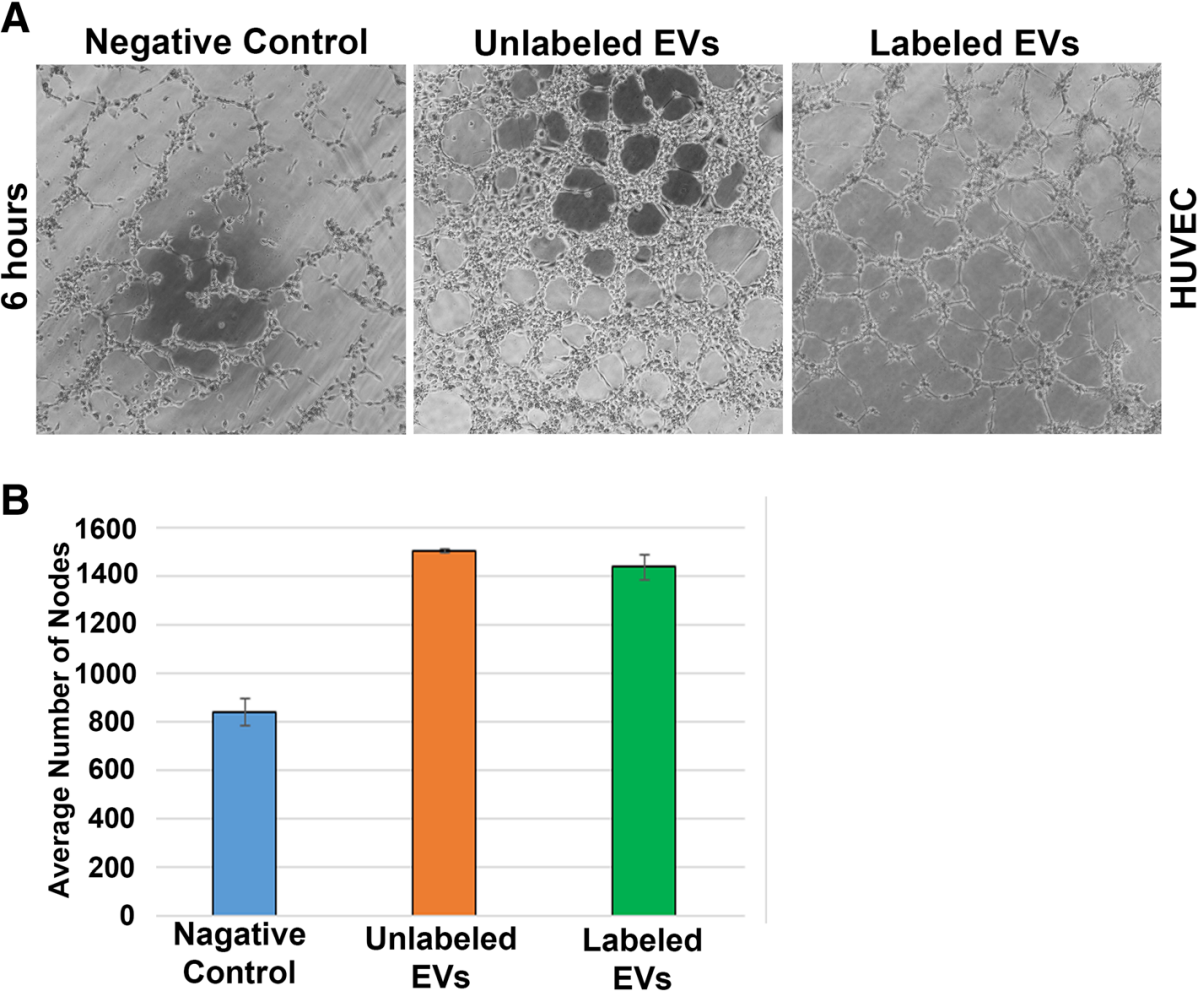
Functionality assay for EVs in HUVECs. Phase contrast images of tube formation assay performed in HUVECs treated with EVs. Cells without EVs served as negative control. The tube forming potential was compared in labeled EVs (primary antibody-TSG101) vs unlabeled EVs (without primary antibody) in HUVECs (**a**). Analyses of the formation of tube nodes in all the three different conditions (**b**)

The role of glioma derived EVs is widely studied. To determine, whether the angiogenic potential of EVs is retained after labeling of EVs using our method, we performed the tube formation assay using HUVECs. The number of nodes formed were calculated using AngioTool64 version 0.6a (02.18.14) under similar software settings like vessel diameter and intensity [26]. As shown by the phase contrast micrographs (Fig. 6a) and the quantified data (Fig. 6b), the labeled EVs were as effective as unlabeled EVs in inducing angiogenesis as compared to HUVECs incubated without EVs.

## Discussion

Isolation of EVs is the most important step for EV research. EVs isolated by ultracentrifugation are often contaminated with other non-vesicular proteins [30]. Antibody based EV isolation is expensive and limited to only a few subpopulations of EVs [31]. Even, size-exclusion chromatography is not able to eliminate lipoprotein contamination. Instead, PEG is a widely used reagent for EV isolation [24, 32, 33]. There are several polymer-based (PEG, Dextran etc.) commercial products which are employed for EV isolation [34]. Here, we have followed a PEG-based protocol for obtaining EVs [24, 34]. PEG helps to precipitate EVs very efficiently, with a higher yield (get visible pellet) and takes lesser amount of time as compared to ultracentrifugation. Here, we have reported development of an efficient antibody-based method for visualizing molecules expressed specifically by EVs isolated from the conditioned medium using PEG followed by size-exclusion column to eliminate the background noise.

Labeling of EVs is very difficult because of their nano-size. Most of the available strategies use surface proteins such as CD63, CD9, CD81 or lipophilic dyes like PKH or Did for labeling of EVs. To our knowledge, there are no efficient protocols available for labeling intra-vesicular proteins like TSG101, HSP70 etc. The method developed by us is one of the unique method for labeling proteins in target EVs. Importantly, it can be employed for labeling of proteins localized either to the surface or present inside the vesicles. Furthermore, our method is perfectly adaptable to situations wherein there is extensive EV heterogeneity.

The existence of heterogeneity within the EVs is a natural phenomenon and is indicative of presence of diverse signaling mechanisms occurring due to intercellular communication arising through EVs [35]. To capture EV-mediated (in terms of molecule content) signaling through their cargo molecules, we essentially require specific and sensitive methods. The method reported here provides an opportunity to label particular population of EVs and thus may pave way for further sorting of this population through nanoscale flow cytometry [10, 36, 37]. Hence, the study on particular population of EVs is easier with our method and we can further analyze the biomolecules carried in these vesicles upon sorting followed by mass spectrometry and RNA-sequencing [36, 37].

We have developed a very unified method for EV labeling which can be further used for confocal, STED and TEM imaging. Our STED microscopy data gives size distribution of about 60 to 400 nm which further supports the existing literature [2, 3]. The applicability of this developed procedure for EVs with diameter greater than 450 nm is not analyzed by us. However, we believe that it may also be possible to label larger EVs using our protocol. Our method eliminates background noise very efficiently; so the gold labeling of particular type of proteins present in EVs becomes easier for visualization by TEM. Our TEM data has confirmed the integrity of EVs during sample preparation and shown that it can be employed for EVs ranging in size from 27 to 230 nm.

Another advantage of our method is that it does not require any intensive cloning strategy or any special microfluidic device for the staining of EVs. We have also used floating EVs in a microfuge tube to reduce the cost of the labeling procedure. Moreover, dual labeling of EV with both antibody and PKH further confirms the specificity of the fluorescent signal.

In conclusion, the method reported here has multiple applications in EV research. The detection of EVs from bio-fluids may also be performed using this method. This would thus help significantly in prognostication, diagnosis and staging of tumors as well as in basic research to understand the functionality of EVs from normal and tumor cells.

## Abbreviations

CFSE: Carboxyfluorescein succinimidyl ester
dSTORM: Direct stochastic optical reconstruction microscopy
EV: Extracellular vesicle
GB: Glioblastoma
GFP: Green fluorescent protein
HSP70: Heat shock protein70
IF: Immunofluorescence
PBS: Phosphate buffer saline
PEG: Polyethylene glycol
RFP: Red fluorescent protein
SEA: Single EV analysis
SEM: Standard error mean
STED: Stimulated emission depletion
TSG101: Tumor susceptibility gene 101 protein
Did: DiIC18(5) solid (1,1'′’-dioctadecyl-3,3,3'′’,3'′’-tetramethylindodicarbocyanine, 4 chlorobenzenesulfonate salt) dye

## References

[1] Lee Y, El Andaloussi S, Wood MJ (2012). Exosomes and microvesicles: extracellular vesicles for genetic information transfer and gene therapy. Hum Mol Genet.

[2] Raposo G, Stoorvogel W (2013). Extracellular vesicles: exosomes, microvesicles, and friends. J Cell Biol.

[3] Colombo M, Raposo G, Théry C (2014). Biogenesis, secretion, and intercellular interactions of exosomes and other extracellular vesicles. Annu Rev Cell Dev Biol.

[4] Minciacchi VR, Freeman MR, Di Vizio D (2015). Extracellular vesicles in cancer: exosomes, microvesicles and the emerging role of large oncosomes. Semin Cell Dev Biol.

[5] Yáñez-Mó M, Siljander PR-M, Andreu Z, Zavec AB, Borràs FE, Buzas EI, Buzas K, Casal E, Cappello F, Carvalho J, Colás E, Cordeiro-da Silva A, Fais S, Falcon-Perez JM, Ghobrial IM, Giebel B, Gimona M, Graner M, Gursel I, Gursel M, Heegaard NHH, Hendrix A, Kierulf P, Kokubun K, Kosanovic M, Kralj-Iglic V, Krämer-Albers E-M, Laitinen S, Lässer C, Lener T, Ligeti E, Linē A, Lipps G, Llorente A, Lötvall J, Manček-Keber M, Marcilla A, Mittelbrunn M, Nazarenko I, Nolte-‘t ENM, Hoen TA, Nyman LO, Driscoll M, Olivan C, Oliveira É, Pállinger HA, Del Portillo J, Reventós M, Rigau E, Rohde M, Sammar F, Sánchez-Madrid N, Santarém K, Schallmoser MS, Ostenfeld W, Stoorvogel R, Stukelj SG, Van der Grein MH, Vasconcelos MHM, Wauben ODW (2015). Biological properties of extracellular vesicles and their physiological functions. J Extracell Vesicles.

[6] Becker A, Thakur BK, Weiss JM, Kim HS, Peinado H, Lyden D (2016). Extracellular vesicles in Cancer: cell-to-cell mediators of metastasis. Cancer Cell.

[7] Mondal A, Kumari Singh D, Panda S, Shiras A (2017). Extracellular vesicles as modulators of tumor microenvironment and disease progression in glioma. Front Oncol.

[8] Qiu J, Yang G, Feng M, Zheng S, Cao Z, You L, Zheng L, Zhang T, Zhao Y (2018). Extracellular vesicles as mediators of the progression and chemoresistance of pancreatic cancer and their potential clinical applications. Mol Cancer.

[9] Grange C, Tapparo M, Collino F, Vitillo L, Damasco C, Deregibus MC, Tetta C, Bussolati B, Camussi G (2011). Microvesicles released from human renal cancer stem cells stimulate angiogenesis and formation of lung premetastatic niche. Cancer Res.

[10] Morales-Kastresana A, Telford B, Musich TA, McKinnon K, Clayborne C, Braig Z, Rosner A, Demberg T, Watson DC, Karpova TS, Freeman GJ, DeKruyff RH, Pavlakis GN, Terabe M, Robert-Guroff M, Berzofsky JA, Jones JC (2017). Labeling extracellular vesicles for nanoscale flow cytometry. Sci Rep.

[11] GRANGE C, TAPPARO M, BRUNO S, CHATTERJEE D, QUESENBERRY PJ, TETTA C, CAMUSSI G (2014). Biodistribution of mesenchymal stem cell-derived extracellular vesicles in a model of acute kidney injury monitored by optical imaging. Int J Mol Med.

[12] Hoshino A, Costa-Silva B, Shen T-L, Rodrigues G, Hashimoto A, Tesic Mark M, Molina H, Kohsaka S, Di Giannatale A, Ceder S, Singh S, Williams C, Soplop N, Uryu K, Pharmer L, King T, Bojmar L, Davies AE, Ararso Y, Zhang T, Zhang H, Hernandez J, Weiss JM, Dumont-Cole VD, Kramer K, Wexler LH, Narendran A, Schwartz GK, Healey JH, Sandstrom P, Jørgen Labori K, Kure EH, Grandgenett PM, Hollingsworth MA, de Sousa M, Kaur S, Jain M, Mallya K, Batra SK, Jarnagin WR, Brady MS, Fodstad O, Muller V, Pantel K, Minn AJ, Bissell MJ, Garcia BA, Kang Y, Rajasekhar VK, Ghajar CM, Matei I, Peinado H, Bromberg J, Lyden D (2015). Tumour exosome integrins determine organotropic metastasis. Nature..

[13] Peinado H, Alečković M, Lavotshkin S, Matei I, Costa-Silva B, Moreno-Bueno G, Hergueta-Redondo M, Williams C, García-Santos G, Ghajar CM, Nitadori-Hoshino A, Hoffman C, Badal K, Garcia BA, Callahan MK, Yuan J, Martins VR, Skog J, Kaplan RN, Brady MS, Wolchok JD, Chapman PB, Kang Y, Bromberg J, Lyden D (2012). Melanoma exosomes educate bone marrow progenitor cells toward a pro-metastatic phenotype through MET. Nat Med.

[14] Lai CP, Kim EY, Badr CE, Weissleder R, Mempel TR, Tannous BA, Breakefield XO (2015). Visualization and tracking of tumour extracellular vesicle delivery and RNA translation using multiplexed reporters. Nat Commun.

[15] Pužar Dominkuš P, Stenovec M, Sitar S, Lasič E, Zorec R, Plemenitaš A, Žagar E, Kreft M, Lenassi M (2018). PKH26 labeling of extracellular vesicles: characterization and cellular internalization of contaminating PKH26 nanoparticles. Biochim Biophys Acta Biomembr.

[16] Takov K, Yellon DM, Davidson SM (2017). Confounding factors in vesicle uptake studies using fluorescent lipophilic membrane dyes. J Extracell Vesicles..

[17] Garcia NA, Moncayo-Arlandi J, Sepulveda P, Diez-Juan A (2016). Cardiomyocyte exosomes regulate glycolytic flux in endothelium by direct transfer of GLUT transporters and glycolytic enzymes. Cardiovasc Res.

[18] Rappa G, Santos MF, Green TM, Karbanová J, Hassler J, Bai Y, Barsky SH, Corbeil D, Lorico A (2017). Nuclear transport of cancer extracellular vesicle-derived biomaterials through nuclear envelope invagination-associated late endosomes. Oncotarget..

[19] Morton MC, Neckles VN, Seluzicki CM, Holmberg JC, Feliciano Correspondence DM (2018). Neonatal subventricular zone neural stem cells release extracellular vesicles that act as a microglial morphogen data and software availability GSE110892. Cell Rep.

[20] Meyer C, Losacco J, Stickney Z, Li L, Marriott G, Lu B (2017). Pseudotyping exosomes for enhanced protein delivery in mammalian cells. Int J Nanomedicine.

[21] Lee K, Fraser K, Ghaddar B, Yang K, Kim E, Balaj L, Chiocca EA, Breakefield XO, Lee H, Weissleder R (2018). Multiplexed profiling of single extracellular vesicles. ACS Nano.

[22] Koliha N, Wiencek Y, Heider U, Jüngst C, Kladt N, Krauthäuser S, Johnston ICD, Bosio A, Schauss A, Wild S (2016). A novel multiplex bead-based platform highlights the diversity of extracellular vesicles. J Extracell Vesicles..

[23] Sharma A, Bendre A, Mondal A, Muzumdar D, Goel N, Shiras A (2017). Angiogenic gene signature derived from subtype specific cell models segregate proneural and mesenchymal glioblastoma. Front Oncol.

[24] Weng Y, Sui Z, Shan Y, Hu Y, Chen Y, Zhang L, Zhang Y (2016). Effective isolation of exosomes with polyethylene glycol from cell culture supernatant for in-depth proteome profiling. Analyst..

[25] Guo S, Lok J, Liu Y, Hayakawa K, Leung W, Xing C, Ji X, Lo EH (2014). Assays to examine endothelial cell migration, tube formation, and gene expression profiles. Methods Mol Biol.

[26] Zudaire E, Gambardella L, Kurcz C, Vermeren S (2011). A computational tool for quantitative analysis of vascular networks. PLoS One.

[27] Schindelin J, Arganda-Carreras I, Frise E, Kaynig V, Longair M, Pietzsch T, Preibisch S, Rueden C, Saalfeld S, Schmid B, Tinevez J-Y, White DJ, Hartenstein V, Eliceiri K, Tomancak P, Cardona A (2012). Fiji: an open-source platform for biological-image analysis. Nat Methods.

[28] Bronisz A, Wang Y, Nowicki MO, Peruzzi P, Ansari KI, Ogawa D, Balaj L, De Rienzo G, Mineo M, Nakano I, Ostrowski MC, Hochberg F, Weissleder R, Lawler SE, Chiocca EA, Godlewski J (2013). Extracellular Vesicles Modulate the Glioblastoma Microenvironment via a Tumor Suppression Signaling Network Directed by miR-1.

[29] Treps L, Perret R, Edmond S, Ricard D, Gavard J (2017). Glioblastoma stem-like cells secrete the pro-angiogenic VEGF-A factor in extracellular vesicles. J Extracell Vesicles.

[30] Coumans FAW, Brisson AR, Buzas EI, Dignat-George F, Drees EEE, El-Andaloussi S, Emanueli C, Gasecka A, Hendrix A, Hill AF, Lacroix R, Lee Y, van Leeuwen TG, Mackman N, Mäger I, Nolan JP, van der Pol E, Pegtel DM, Sahoo S, Siljander PRM, Sturk G, de Wever O, Nieuwland R (2017). Methodological guidelines to study extracellular vesicles. Circ Res.

[31] Furi I, Momen-Heravi F, Szabo G (2017). Extracellular vesicle isolation: present and future. Ann Transl Med.

[32] Andreu Z, Rivas E, Sanguino-Pascual A, Lamana A, Marazuela M, González-Alvaro I, Sánchez-Madrid F, de la Fuente H, Yáñez-Mó M (2016). Comparative analysis of EV isolation procedures for miRNAs detection in serum samples. J Extracell Vesicles..

[33] Konoshenko MY, Lekchnov EA, Vlassov AV, Laktionov PP (2018). Isolation of extracellular vesicles: general methodologies and latest trends. Biomed Res Int.

[34] Rider MA, Hurwitz SN, Meckes DG (2016). ExtraPEG: a polyethylene glycol-based method for enrichment of extracellular vesicles. Sci Rep.

[35] Willms E, Cabañas C, Mäger I, Wood MJA, Vader P (2018). Extracellular vesicle heterogeneity: subpopulations, isolation techniques, and diverse functions in Cancer progression. Front Immunol.

[36] Poon AC, Garzon J, Brett S, Lowerison M, Williams K, Leong HS. Nanoscale flow cytometry of patient plasma for the detection of prostate cancer-associated extracellular vesicles. Biochemistry. 2017. 10.17975/sfj-2017-010.

[37] Zhu S, Ma L, Wang S, Chen C, Zhang W, Yang L, Hang W, Nolan JP, Wu L, Yan X (2014). Light-scattering detection below the level of single fluorescent molecules for high-resolution characterization of functional nanoparticles. ACS Nano.

